# Difficulties that unexpected results face to be accepted: suicide and the moon

**DOI:** 10.1038/s41380-021-01028-x

**Published:** 2021-02-01

**Authors:** V. Benno Meyer-Rochow, Tapani Hakko, Helinä Hakko, Pirkko Riipinen, Markku Timonen

**Affiliations:** 1 Department of Plant Medicals, Agricultural Science and Technology Institute, Andong National University, Andong, GB36729 Republic of Korea; 2 Department of Ecology and Genetics, University of Oulu, Oulu, Finland; 3 Department of Psychiatry, Oulu University Hospital, Oulu, Finland; 4 Research Unit of Clinical Neuroscience, Psychiatry, Oulu, Finland; 5 Center of Life Course Health Research, University of Oulu, Oulu, Finland; 6 Unit of General Practice, Oulu University Hospital, Oulu, Finland

**Keywords:** Diseases, Psychiatric disorders

## To the Editor:

It is admirable that Plöderl and Hengartner have taken up the task to examine publications for occurrences of false positives and other flaws [1, 2]. That is a major undertaking and will likely occupy them for many years to come since in their own words “most research findings are false positives”. In psychiatric publications successful replications were in fact possible only in one third of the published cases [3]. The question is whether the other two thirds were useless articles, especially in view of the fact that in epidemiological studies data are usually gathered for several purposes and compromises are necessary to decide what to gather and what to ignore. Consequently, epidemiological data may, to some extent, always have some limitations.

It is correct that we did not discuss the possibility of a false-positive finding. However, since none of the three reviewers of the original manuscript expressed a need for such a discussion, we did not include an analysis on false-positive results. Plöderl and Hengartner are also correct in stating that our results are novel in view of “given what we know about the lack of association between moon phases and suicide” (Plöderl and Hengartner). However, is what has been widely believed until now about moon phases and suicides actually true?

Lunar phases are well known to affect reproductive and other behaviours in animals, but were dismissed for years in connection with humans until Wehr [4] could recently show that synchrony of sleep-wake and bipolar mood cycles can indeed be related to lunar cycles. Earlier rejected as false, earthquakes have also recently been shown to be associated with lunar phases [5], and contrary to earlier assumptions, homicides (at least in Finland) were now found to be moon-phase correlated [6]. In relation to suicides, to dismiss the studies with positive findings, cited in [7], as “may also be false positives” by Plöderl and Hengartner, assumes that our study was false-positive (for which there is no clear proof on account of the differences in geographic and social setting between other studies as Plöderl and Hengartner themselves admit in the penultimate sentence of their Commentary).

It is necessary to guard against a well-documented reluctance to embrace novel suggestions, ideas and findings that depart from convention. The hesitation to accept new ideas in medicine has even been given the name “Semmelweis Reflex” [8] after the Hungarian doctor, who intuited the iatrogenic and infectious causes of puerperal fever and then was violently opposed for questioning old dogmas. A similar fate of initial rejections, because the new findings challenged accepted paradigms, was experienced in connection with the discovery by Marshall and Warren that stomach ulcers were caused by an organism, namely *Helicobacter pylori*, and by the finding of Mourtzoukou and Falagas who showed that feeling cold increased the susceptibility to infections [9]. Now fully accepted, none of these studies mentioned above examined the possibility of false-positives.

The value of our study is that it encourages suicide researchers to take a more differentiated look at suicide victims and the timing of their suicides and to examine premenstrual and postmenstrual female victims separately. This has almost never been done in the past and in view of our results may now need to become something to be considered more routinely by suicide researchers. Although we did not include the statistical data, we did mention in the results the marginally significant finding that a history of hospital-treated psychiatric disorders was recorded in full moon suicides of women. The discussion about the role of seasonal depression in our paper is therefore justified and to analyze the seasonal proportions of psychiatric disorders of suicide victims and moon phases in the future could be interesting.

We close this commentary with a reference to an editorial with the title “Rationalisation of Scientific Publication” that was published in a 1935 Nature issue [10] and which is as pertinent today as it was then: “Conservatism can be preserved from inertia and ineptitude only by judgement and the assimilation, not the rejection, of new ideas”.
